# Evaluation of aortic stenosis using cardiovascular magnetic resonance: a systematic review & meta-analysis

**DOI:** 10.1186/s12968-020-00633-z

**Published:** 2020-06-15

**Authors:** Kei Woldendorp, Paul G. Bannon, Stuart M. Grieve

**Affiliations:** 1 Sydney Translational Imaging Laboratory, Imaging and Phenotyping Laboratory, Charles Perkins Centre, Faculty of Medicine and Health, The University of Sydney, Camperdown, NSW 2006 Australia; 2 Sydney Medical School, The University of Sydney, Camperdown, NSW 2050 Australia; 3 Baird Institute of Applied Heart & Lung Surgical Research, Newtown, NSW 2042 Australia; 4 Department of Cardiothoracic Surgery, Royal Prince Alfred Hospital, Camperdown, NSW 2006 Australia; 5 Department of Radiology, Royal Prince Alfred Hospital, Camperdown, NSW 2006 Australia

**Keywords:** Cardiovascular magnetic resonance, CMR, Aortic valve, Aortic stenosis, Aortic regurgitation, Valve dysfunction

## Abstract

**Background:**

As the average age of patients with severe aortic stenosis (AS) who receive procedural intervention continue to age, the need for non-invasive modalities that provide accurate diagnosis and operative planning is increasingly important. Advances in cardiovascular magnetic resonance (CMR) over the past two decades mean it is able to provide haemodynamic data at the aortic valve, along with high fidelity anatomical imaging.

**Methods:**

Electronic databases were searched for studies comparing CMR to transthoracic echocardiography (TTE) and transoesophageal echocardiography (TEE) in the diagnosis of AS. Studies were included only if direct comparison was made on matched patients, and if diagnosis was primarily through measurement of aortic valve area (AVA).

**Results:**

Twenty-three relevant, prospective articles were included in the meta-analysis, totalling 1040 individual patients. There was no significant difference in AVA measured as by CMR compared to TEE. CMR measurements of AVA size were larger compared to TTE by an average of 10.7% (absolute difference: + 0.14cm^2^, 95% CI 0.07–0.21, *p* < 0.001). Reliability was high for both inter- and intra-observer measurements (0.03cm^2^ +/− 0.04 and 0.02cm^2^ +/− 0.01, respectively).

**Conclusions:**

Our analysis demonstrates the equivalence of AVA measurements using CMR compared to those obtained using TEE. CMR demonstrated a small but significantly larger AVA than TTE. However, this can be attributed to known errors in derivation of left ventricular outflow tract size as measured by TTE. By offering additional anatomical assessment, CMR is warranted as a primary tool in the assessment and workup of patients with severe AS who are candidates for surgical or transcatheter intervention.

## Introduction

Cardiovascular magnetic resonance imaging (CMR) has, since its introduction in the 1980s, evolved to become a viable non-invasive alternative to echocardiography for a wide variety of cardiac pathology. Aortic stenosis (AS) is a common disease with a devastating clinical impact; without intervention AS progresses inexorably and once symptoms develop the life-expectancy is reduced to an average of 3 years unless the mechanical obstruction is not relieved [1]. Transthoracic echocardiography (TTE) is the clinical reference standard - it is rapid, safe, well tolerated by patients and is by far the most common exam used for evaluation of aortic valvular disease. The considerable technical advances in CMR have improved the quality of the anatomical and functional information available from CMR. Here, we systematically the current available evidence regarding the use of CMR compared to echocardiography to evaluate the clinical status and practicality of this technique [2].

Diagnosis of AS, and particularly severe AS is determined by a combination of mean and peak pressure gradients across the valve as well as the effective valve orifice, or aortic valve area (AVA) [2]. Based on these criteria severe AS is specified by an AVA of <1cm^2^ determined by the maximum opening of the aortic valve during systole [3]. Several methods have been designed to calculate this, including the Gorlin formula [4] for use with invasive cardiac catheterisation (now seldom used), the continuity equation [5] used with TTE and determined on a series of measurements including the left ventricular outflow tract (LVOT), and planimetry [6] and computed tomography (CT) that determine AVA by direct measurement of the valve orifice.

CMR has emerged as an alternate, non-invasive method, and offers prognostic and planning potential for procedural intervention in the form of transcatheter aortic valve implantation (TAVI) or surgical aortic valve replacement (SAVR). Additionally, CMR provides high fidelity anatomical imaging, thus avoiding the need for additional imaging tools such as CT in the workup for valve intervention. This may be particularly beneficial in patients with impaired renal function where high iodinated contrast loads required for CT imaging are contraindicated [7]. Contrast enhanced CMR can provide valuable information on anatomy and myocardial scar, however gadolinium contrast is not required for basic functional assessment or for valve flow quantification [8].

The rapid recent growth in volume of aortic interventional procedures is increasing demand for accurate assessment of aortic valve anatomy and function. CMR represents an attractive non-invasive method in view of the lack of radiation exposure, the relative safety of non-ionic gadolinium agents relative to iodine contrast, and the ability of CMR to provide a mix of cross-sectional anatomical, flow and ventricular functional information. This paper aims to review the current literature on use of CMR for aortic valve assessment in comparison to both TTE and transesophageal echocardiography (TEE).

## Methods

**Fig. 1:**
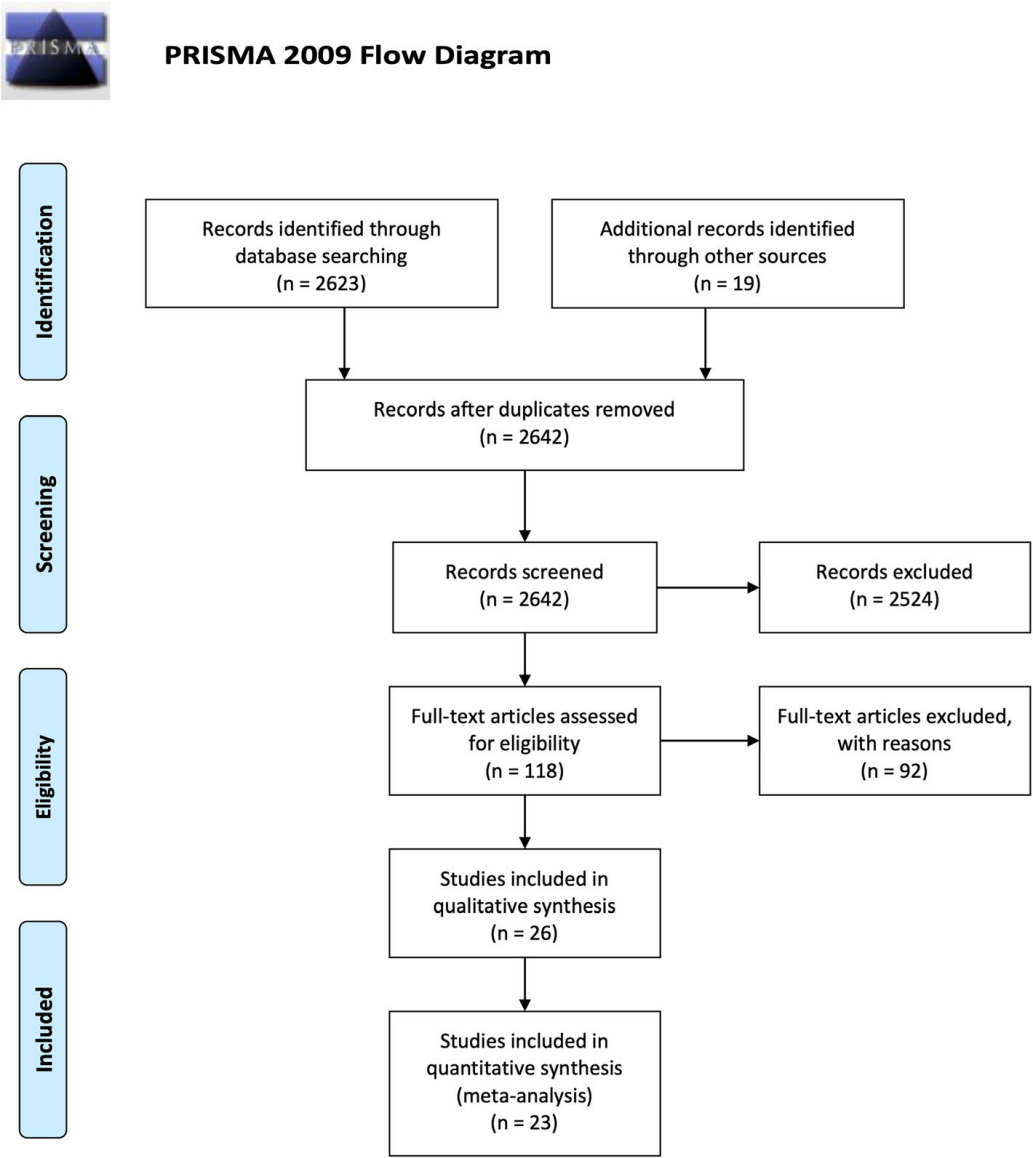
PRISMA search strategy

Following the PRISMA (Preferred Reporting Items for Systematic Reviews and Meta-Analysis) guidelines, electronic searches were conducted by two authors (KW + SG). Electronic searches were performed using Web of Knowledge, Embase, and PubMed. Keywords included in the search were “CMR”, “cardiac magnetic resonance”, “aorta”, “aortic valve” “aortic root” and “aortic pathology”. Eligible studies were prospective studies published in English that compared CMR with either TTE or TEE for AVA evaluation. The reference lists of all retrieved articles were reviewed for further identification of potentially relevant studies (Fig. 1). All data were extracted from article texts, tables, figures, and appendices. Articles included were assessed for quality using the Newcastle-Ottawa Quality Assessment Scale (NOS) (Supplementary).

### Inclusion criteria

Only studies that specified either measured or derived AVA (as opposed to correlations between imaging methods) were included in the meta-analysis portion of the review.

### Analysis

Baseline characteristics and intervention details were presented as raw values (%) or mean ± standard deviation unless otherwise indicated. Pooled values for clinical outcomes were reported as mean ± standard deviation or as otherwise specified. Data were summarized as standard mean difference, with overall weighted mean presented where appropriate. I^2^ statistic was used to estimate the percentage of total variation across studies, due to heterogeneity rather than chance. An I^2^ value of greater than 50% was considered substantial heterogeneity. If there was substantial heterogeneity, the possible clinical and methodological reasons for this were explored qualitatively. All *P*-values were 2-sided. A significant difference was defined as *P* < 0.05 (without correction for multiplicity). Statistical analysis was conducted with Review Manager Version 5.3 (Cochrane Collaboration, Software Update, Oxford, UK).

## Results

A total of 2623 publications were identified through two online database searches and from collated reference lists (Fig. 1). After exclusion of duplicated or irrelevant publications, a total of 23 relevant, prospective articles were included in the meta-analysis, totalling 1040 individual patients [9–31]. One study which investigated the use of CMR in subsets of low flow/low gradient AS (59 patients of 128 total) was separated into four discrete datasets for the meta-analysis [9] (Table 1). AS was identified in 659 (63.4%) patients, aortic regurgitation (AR) in 26 (2.2%), mixed pathology in 74 (6.3%), and controlled subjects numbered 294 (28.3%).

**Table 1:** Summary of papers included in the meta-analysis

					Pathology			AVA (cm^2^)				Reliability (CMR)	
Author	Year	n	Males	Age (yrs)	AS	AR	Mixed	CMR	TTE	TEE		Inter	Intra
Barone-Rochette HG/NF	2013	69	43	75 ± 10	69			1.0 ± 0.02	0.71 ± 0.13				
Barone-Rochette HG/LF	2013	28	16	72 ± 14	28			0.9 ± 0.1	0.54 ± 0.12				
Barone-Rochette LG/NF	2013	17	8	72 ± 9	17			1.2 ± 0.2	0.84 ± 0.09				
Barone-Rochette LG/LF	2013	14	8	72 ± 6	14			1.0 ± 0.2	0.81 ± 0.15				
Buchner	2015	8	1		8			0.75 ± 0.09	0.69 ± 0.07	0.79 ± 0.15		6%	3%
Debl	2005	33						0.94 ± 0.29	0.85 ± 0.31				
Defrance	2012	74	33	75 ± 14	43			1.00 (0.80–2.30)	0.93 (0.68–2.42)			1.80 ± 2.27%	
Dimitriou	2012	14			14			1.0 ± 0.4	1.0 ± 0.2				
Friedrich	2001	25		64 ± 8	15		10	0.86 ± 0.25	0.79 ± 0.2			15.20%	17.80%
Garcia	2013	68	39	64 ± 15	60			1.4 ± 0.41	1.19 ± 0.28				
John	2003	40	25	70 ± 8.8	40			0.91 ± 0.25		0.89 ± 0.28		0.07 ± 0.06	0.05 ± 0.04
Knobelsdorff-Brenkenhoff	2009	65			2	1		1.71 ± 0.46	1.7 ± 0.4	1.82 ± 0.53		11.5 ± 7.8%	6.7 ± 5.4%
Kupfahl	2003	44	27		44			0.8 ± 0.25	0.7 ± 0.3	0.8 ± 0.28		0.03 ± 0.05	− 0.02 ± 0.06
Levy	2016	91	60	74 ± 10	91			0.9 ± 0.22	0.81 ± 0.18			0.83 [0.42–0.95]	0.82 [0.39–0.95]
Malyar	2008	42	17	71 ± 8	20		22	0.97 ± 0.3^a^	0.75 ± 0.28^b^	0.87 ± 0.25^c^			
Mutnuru	2016	50			10	9	8	1.12 ± 0.25	1.10 ± 0.21				
O’Brien	2009	15			15			0.85 ± 0.3		0.85 ± 0.24			
Paelinck	2011	24	8	83.5 (67–88)	24			0.60 (0.3–0.8)	0.54 (0.32–0.83)	0.6 (0.37–0.8)			
Pontone	2013	50	27	79.6 ± 7.5	50			0.4 ± 0.1	0.4 ± 0.1				
Pouleur	2007	48	33	62 ± 13	27			2.4 ± 1.8	2.0 ± 1.5	2.5 ± 1.7		0.1 ± 0.3	0.0 ± 0.3
Reant	2006	39	25	71.1 ± 7.6	13		26	0.92 ± 0.29	0.75 ± 0.28	0.93 ± 0.31		0.03 ± 0.14 cm2	0.02 ± 0.07 cm2
Speiser	2014	48	30	64 ± 18	23			1.9 ± 1.1	1.7 ± 0.8			0.027 ± 0.13 cm2 (0.53%)	0.027 ± 0.06 cm2 (0.69%)
Weininger	2011	22	13		22			0.65 ± 0.34		0.78 ± 0.15		0.01 ± 0.03	0.01 ± 0.02
Westermann	2011	27	16	61.8 ± 8.3	18	1		1.04 ± 0.39^d^	0.88 ± 0.22			4.3 ± 2.6%	2.9 ± 1.0%

*AR* Aortic regurgitation, *AS* Aortic stenosis, *CMR* Cardiovascular magnetic resonance, *HG/NF* High-gradient/normal-flow, *HG/LF* High-gradient/low-flow, *LG/NF* Low-gradient/normal-flow, *LG/LF* Low-gradient/low-flow, *TEE* Transesophageal echocardiography, *TTE* Transthoracic echocardiography

^a^only completed in 26 patients

^b^only completed in 41 patients

^c^only completed in 38 patients

^d^only for patients with AS

The vast majority of measurements were done using a 1.5 T CMR (1030 subjects, 99.1%) patients, a 3 T CMR was used in only 10 (0.9%) patients. CMR protocols used included balanced steady-state free procession (bSSFP) in 967 (93.0%) patients and gradient echo (GRE) in 73 (7.0%) patients. Where multiple protocols were used, the one closest to the study result was used in the meta-analysis, as detailed in the footnotes. Measurements of AVA by CMR was performed by planimetry of the inner valve leaflet edges during maximal opening in systole, this was similar to measurements obtained by TEE. TTE measurements were by derived using the continuity equation.

### Valve area assessment

**Fig. 2:**
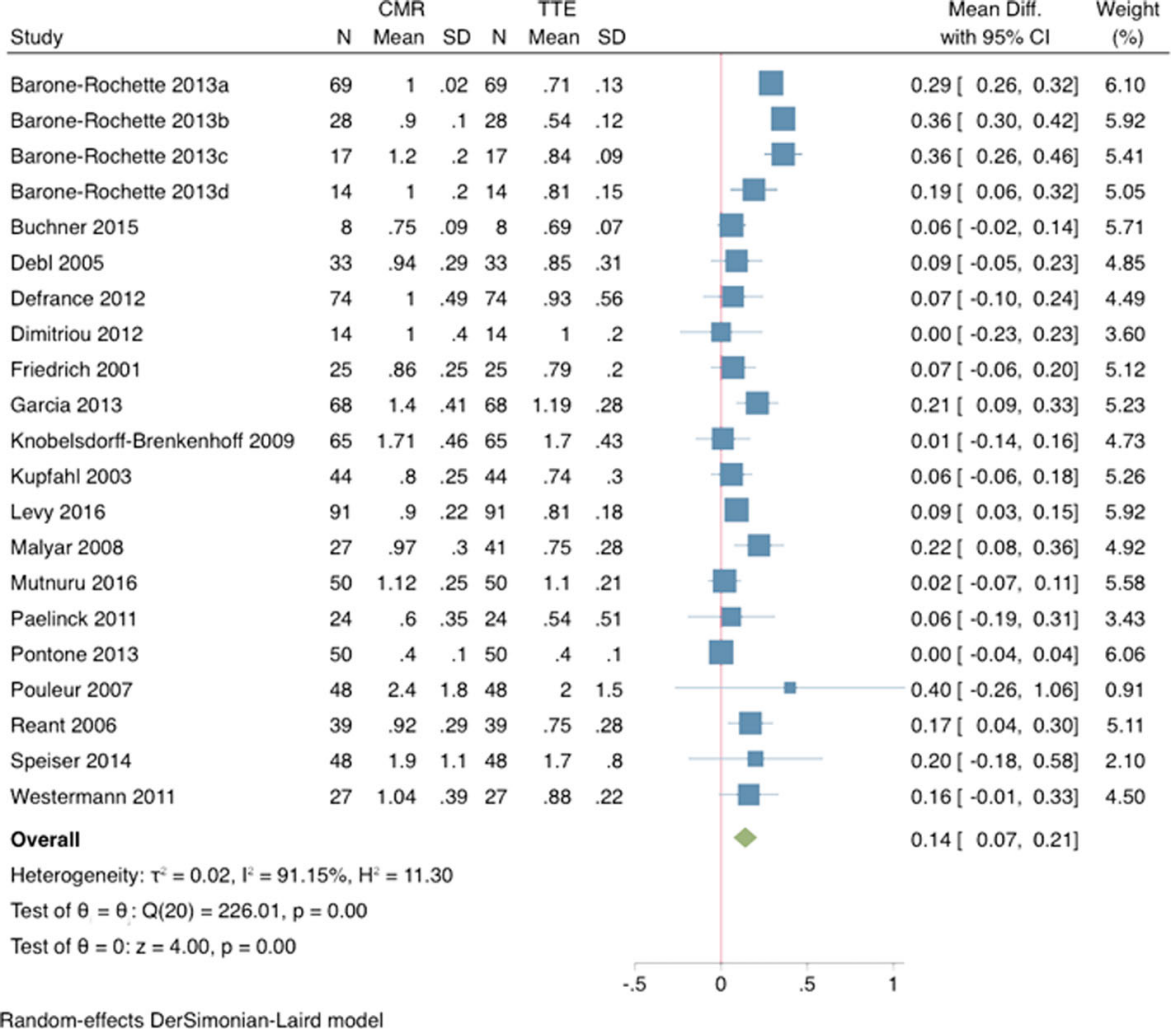
Comparison of cardiovascular magnetic resonance (CMR) to transthoracic echocardiography (TTE) for assessment of aortic valve area (AVA). *Forrest plot of AVA measurements (mean + SD) for CMR and TTE demonstrate a significantly larger measurement obtained by CMR as compared to TTE*

**Fig. 3:**
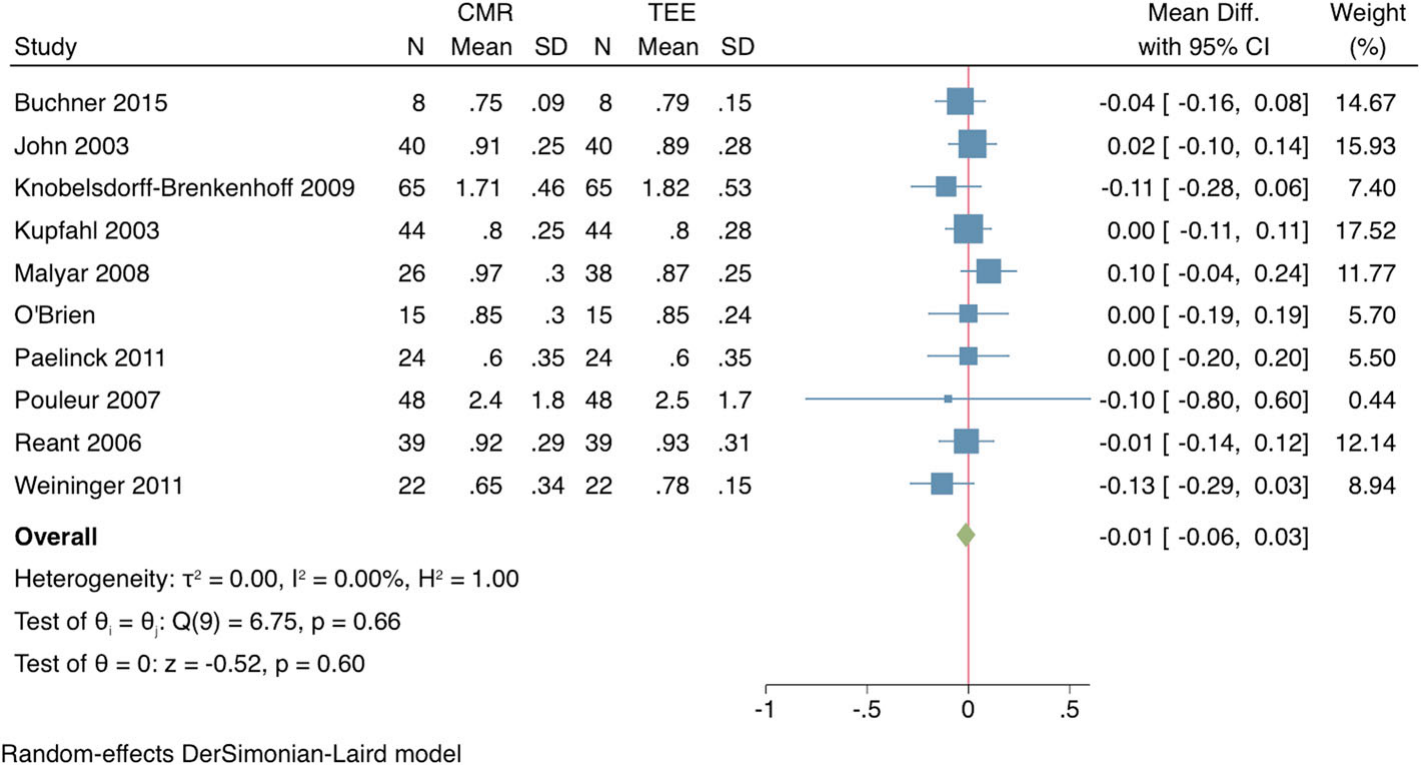
Comparison of CMR to transesophageal echocardiography (TEE) for assessment of AVA. *Forrest plot of AVA measurements (mean + SD) for CMR and TEE demonstrate no significant difference between the two modalities*

Mean CMR measured AVA was significantly larger than TTE by 10.7% (mean difference: + 0.14cm^2^, 95% CI 0.07–0.21, *p* < 0.001), but was equivalent to TEE measurements (− 0.01cm^2^, 95% CI -0.06 – 0.03, *p* = 0.68) (Figs. 2 and 3).

### Repeatability

Table 1 further summarises data from the 14 studies that presented data on either inter- or intra-observer variability. This data was present in a mixture of formats and was therefore not included in the formal meta-analysis. Reliability of CMR was generally high with mean inter-observer difference of 0.03cm^2^ +/− 0.04 (7 studies) and 3.1% +/− 0.35 (7 studies). Similarly, intra-observer difference was also small, 0.02cm^2^ +/− 0.01 (7 studies) and 2.3% +/− 0.22 (7 studies).

### Haemodynamics

Of the papers reviewed for this study six compared haemodynamic measurements between CMR and TTE. CMR techniques were heterogeneous, including some novel algorithms. Overall haemodynamic measurements by CMR demonstrated good correlation to TTE, however absolute values tended to be lower (Table 2).

**Table 2:** Comparison of haemodynamic measurements between CMR and TTE

Author	Year	n	AS	Method	Conclusion
Defrance	2012	74	53	Novel semi-automated process as described by Bollache, et al. 2010	Good agreement with TTE for PV and MG (*r* = 0.92 with mean bias −0.29 ± 0.62 m/s and r = 0.86 with mean bias −12 ± 15 mmHg, respectively)
Caruthers	2002	24	24	Simpsons rule for VTI and modified Bernoulli equation for gradients	Good agreement with TTE for PG and MG (*r* = 0.83 and r = 0.87, respectively)
Levy	2016	91	91	Not described	Good agreement with TTE for PV (*r* = 0.73 with mean bias −0.35 ± 0.40 m/s)
Garcia	2013	68	60	Simplified Bernoulli equation	Good agreement with TTE for MG (*r* = 0.7 with mean bias −2.8 mmHg)
Eichenberger	1993	19	19	Simplified Bernoulli equation	Good agreement with TTE for MG (*r* = 0.96)
Sirin	2014	19^a^	19	Simplified Bernoulli equation	Good agreement with TTE for MG (*r* = 0.84 with mean bias −12.4 mmHg)

^a^Paediatric patients

*PV* Peak velocity, *MG* Mean gradient, *PV* Peak gradient, *VTI* Velocity time interval

## Discussion

This meta-analysis of 1040 patients compared CMR to currently validated diagnostic techniques for estimation of AVA as part of the diagnosis of AS. CMR compared favourably to TEE, with no significant difference in measurements across these modalities. AVA measurements from CMR overestimate those from TTE by an average of 0.17cm^2^.

A standard for measurement of AS is cardiac catheterisation, but due to its invasive nature and risk of potential stroke is rarely performed in favour of less invasive and safer procedures. TTE is the clinical reference standard, however previous studies have shown that CMR performs as well as, or better than, TTE or TEE compared to direct catheterisation in the assessment of aortic stenosis [20, 28].

The AVA measurement from TTE assessment is derived using the continuity equation and is based on the ratio between Doppler stroke volume and post-aortic valve flow. Calculation of Doppler stroke volume relies on an accurate estimation of the LVOT, a value that is then squared in the continuity equation formula. In TTE this is done at one measurement, in the parasternal long-axis view, on the assumption that the LVOT is circular. However experience from TAVI valve sizing highlights that the LVOT is frequently elliptical and thus measurements from TTE may underestimate or underestimate the true LVOT size, and subsequently also underestimate AVA [32, 33] or overestimated AVA. This was confirmed in a paper by Chin et al., which demonstrated that TTE underestimated AVA by 0.23cm^2^ compared with CMR [34]. Similar discrepancies in calculated AVA have been noted in other studies comparing CT to TTE and TEE to TTE for AVA measurements [35, 36]. The discrepancies noted have significant implication on AS grading and provide an explanation for the so-named “paradoxical low-flow, low-gradient severe AS” [34]. Furthermore, use of the continuity equation creates further confoundment in the form of pressure recovery estimation errors of AS severity. In a large cohort study of over 1000 patients it was noted that with specific pressure recovery adjustment almost 50% of severe AS patients were reclassified as non-severe [37]. The ability of CMR to assess complete cardiac function, including blood velocity in multiple sites simultaneously, makes correction of pressure recovery discrepancies far easier and less prone to user error [38, 39].

For patients with low gradient severe aortic stenosis. There was again good correlation between TTE and CMR values for measured LVOT and AVA. CMR calculated AVA slightly larger in patients with low gradient severe aortic stenosis compared to high gradient severe AS, however as mentioned above this is likely due to estimation errors derived from TTE [9]. This highlights a major advantage of CMR over echocardiography for anatomical based diagnosis of patients with low flow low gradient severe aortic stenosis.

Patients with severe AS who are TAVI candidates need accurate anatomical assessments of their LVOT, aortic annulus size, coronary anatomy, and ascending aorta. Studies have demonstrated that CMR produces values in keeping with those seen in CT and TEE for morphological assessments [26, 40, 41]. CT is able to provide precise evaluation of vascular and annular calcification, as well as routine vessel size measurements - facilitating interventional planning. CMR could potentially be used similarly but this is not currently widely practiced. However CMR is the gold standard for functional evaluation of the left ventricle and, unlike CT, can identify the pathological effects of left ventricular remodelling, particularly subendocardial fibrosis, which have been implicated in prognosis post AVR or TAVI [30, 42].

CMR allows for more accurate assessment of valve pathology because it can render images in any plane, however this comes at the price of some loss finer detail due to thicker imaging slices in lower powered machines [43]. Nevertheless, studies have demonstrated higher sensitivity and specificity of CMR to identify correct valvular pathology when compared with traditional methods and confirmed pathologically [11, 44, 45]. Because of the ability of CMR to measure time-resolved, cross-sectional flow (as opposed to inferred flow rates derived in Doppler echocardiography) it may also provide benefit in patients with complex aortic morphology not amenable to echocardiographic assessment [43].

CMR also avoids many of the pitfalls of other imaging techniques including: unnecessary sedation/anaesthesia for TEE and contrast exposure of CT. Gadolinium contrast is not necessary for evaluation of function using cine CMR sequences or for quantification of flow using phase contrast techniques. Avoiding the contrast loading of CT is often cited as another potential benefit of CMR. Currently guidelines still advocate caution and most suggest an estimated glomerular filtration rate (eGFR) < 30 ml/min/1.73 m^2^ be a cut-off for patients who are at increased risk of contrast induced nephropathy [7]. Considering that most patients with severe AS are elderly, and eGFR has a natural decline with age, this presents significant implications to anatomical screening in prospective patients with iodine contrast reliant methods.

CMR has the potential for accurate haemodynamic measurements of the cardiovascular system [8, 46–48]. However, this is not standard practice and significant heterogeneity exists in the algorithms used to calculate transvalvular aortic gradients. The prevailing evidence in the literature suggests that current clinical CMR protocols give reasonably good correlation to TTE haemodynamic measurements but in general will underestimate these to a variable degree [13, 17, 21, 49, 50]. The presence of turbulent flow present challenges for both echocardiography and CMR measurements and accounts for some differences between [17]. Turbulent flow results in signal loss during CMR due to intravoxel dephasing, which may lead to inaccurate measurements of effective orifice area boundaries [51]. Peak gradient estimation in echocardiography is directly affected by turbulent flow since the Bernouli equation can underestimate the true pressure difference by not accounting for flow turbulence, and overestimate the total pressure loss by neglecting the effect of pressure recovery in the post-stenotic region [17, 52]. CMR evaluation of flow at extremely high flow is known to underestimate velocity measurements, secondary to intravoxel dephasing errors [25], although improvements in CMR hardware have improved performance through reductions in echo time (TE) and secondary eddy currents [53]. Advanced methods such as adaptive valve plane phase-contrast CMR and 4D-flow CMR are now clinically feasible, but have not yet translated to the clinical setting, and will need clinicals familiar to their protocols to become used in general clinical applications.

## Conclusion

Our analysis demonstrates the equivalence of valve area measurements in AS using CMR compared to those obtained using TEE. A small but significance difference was noted between CMR and TTE, where higher values found using CMR. Prior data shows that TTE underestimates AVA calculated from LVOT area compared to TEE, attributed to incomplete visualisation of the non-uniform anatomy of the aortic valve. Evidence relating to aortic velocity and pressure gradient measurements are highly heterogeneous, reflecting the complex and evolving nature of CMR flow technology. Given the equivalence of CMR AVA to the clinical reference standard, the additional benefits of CMR may increase the value proposition of this technique for pre-operative workup. Although TTE is rapid, safe, and well tolerated by patients, the ability of CMR to accurately image the remainder of the cardiovascular system, warrant its inclusion in the broader assessment and surgical workup of patients with AS.

## Data Availability

Not applicable.

## Abbreviations

AR: Aortic regurgitation
AS: Aortic stenosis
AVA: Aortic valve area
bSSFP: Balanced steady state free precession
CMR: Cardiovascular magnetic resonance
CT: Computed tomography
eGFR: Estimated glomerular filtration rate
LVOT: Left ventricular outflow tract
MG: Mean gradient
NOS: Newcastle-Ottawa Quality Assessment scale
PG: Peak gradient
PRISMA: Preferred Reporting Items for Systematic Reviewrs and Meta-Analysis
PV: Peak velocity
SAVR: Surgical aortic valve replacement
TAVI: Transcatheter aortic valve implantation
TE: Echo time
TEE: Transesophageal echocardiography
TTE: Transthoracic echocardiography
